# Association between microsatellite status and characteristics and outcomes of early-onset compared to late-onset rectal cancer

**DOI:** 10.1007/s00384-024-04604-z

**Published:** 2024-03-28

**Authors:** Sameh Hany Emile, Nir Horesh, Zoe Garoufalia, Rachel Gefen, Peige Zhou, Victor Strassmann, Steven D. Wexner

**Affiliations:** 1 Ellen Leifer Shulman and Steven Shulman Digestive Disease Center, Cleveland Clinic Florida, 2950 Cleveland Clinic Blvd., Weston, FL 33179 USA; 2 Colorectal Surgery Unit, General Surgery Department, Mansoura University Hospitals, Mansoura, Egypt; 3 Department of Surgery and Transplantation, Sheba Medical Center, Ramat-Gan, Israel; 4 Department of General Surgery, Faculty of Medicine, Hadassah Medical Organization, Hebrew University of Jerusalem, Jerusalem, Israel

**Keywords:** Microsatellite status; Rectal cancer; Age, Survival, Early-onset

## Abstract

**Background:**

Microsatellite instability (MSI) is an important prognosticator for colorectal cancer (CRC). The present study aimed to assess the impact of MSI status on the characteristics and outcomes of early-onset compared to late-onset rectal cancer.

**Methods:**

This retrospective cohort study used data from the US National Cancer Database (2004–2019) to assess the baseline characteristics, treatment patterns, short-term outcomes, and overall survival (OS) of early-onset rectal adenocarcinoma affecting patients < 50 years compared to late-onset rectal adenocarcinoma according to the MSI status.

**Results:**

The present study included 48,407 patients (59.9% male) with rectal cancer, 17.3% of patients were < 50 years and 6.3% had MSI-H tumors. In the early-onset group, patients with MSI-H tumors had a lower mean age (41.5 vs 43 years, *p* < 0.001) and presented less often with stage IV disease (22.1% vs 17.7%, *p* = 0.03) and liver metastasis (9.1% vs 13.5%, *p* = 0.011) than patients with MSS tumors. In the late-onset group, patients with MSI-H and MSS tumors had similar demographics, disease stage, and metastatic pattern, yet MSI-H patients more often received neoadjuvant radiation therapy (58.9% vs 55.1%, *p* = 0.009) and neoadjuvant systemic therapy (40% vs 36.2%, *p* = 0.005). In both age groups, MSI-H tumors were associated with more pathologic T3-4 stage and were more likely mucinous and poorly differentiated carcinomas than MSS tumors. The median OS of MSI-H tumors was similar to MSS tumors (108.09 vs 102.31 months, *p* = 0.1), whether in the early-onset (139.5 vs 134.2 months, *p* = 0.821) or late-onset groups (106.1 vs 104.3 months, *p* = 0.236).

**Conclusions:**

In both age groups, MSI-H rectal cancers were more often mucinous and poorly differentiated carcinomas and had pT3-4 stage more often than MSS cancers. MSI-H rectal cancers tend to present less often with distant metastases and nodal involvement than MSS cancers only in early-onset, but not in late-onset rectal cancers. The association between MSI status and survival was not notable in this study, whether in the early-onset or late-onset groups.

## Introduction

Traditionally, colorectal cancer (CRC) is known to be a disease that affects patients above 50 years of age. However, CRC can affect younger patients, either as a hereditary condition or on a sporadic basis. A concerning rise in the incidence of early-onset colorectal cancer (CRC) in people younger than 50 years has been recently observed [1] which led to reduction of the starting age of screening colonoscopy in some guidelines to 45 years [2]. Early-onset CRC is a distinct entity with different clinicopathologic and molecular features from those of late-onset CRC [3]. While the exact mechanism of the atypical onset of CRC in younger people is unknown, some authors attribute it to Westernized diet, obesity, and alterations in the gut microbiome [4]. It was noted that early-onset CRC has a predilection to affect the left-sided colon and rectum more than the proximal large bowel, usually presents in a more advanced stage, and has unfavorable pathological features. However, it has comparable oncologic outcomes and survival to late-onset CRC [5, 6].

One of the important molecular features that was found to impact survival in CRC is the microsatellite status. Microsatellite instability (MSI) is found in up to 15% of CRC and is attributable to dysfunction of mismatch repair (MMR) genes. Microsatellites are repetitive errors in DNA sequences secondary to failed repair [7]. When microsatellite testing demonstrates mutations in 30% or more microsatellites, the tumor is defined as microsatellite instability-high (MSI-H). It was found that MSI-high CRC responds differently to adjuvant systemic therapy and has an improved stage-adjusted survival compared with microsatellite stable (MSS) CRC [8].

A previous collaborative study [9] looked at the impact of microsatellite status in early-onset colon cancer and observed that 26.2% of patients younger than 50 years had MSI-H colonic cancer that mainly affected the proximal colon. MSI-H colon cancer in young patients had more tumor budding, KRAS mutations, BRAF mutations, and nodal metastases than older patients with MSI-H cancers. The 5-year disease-free survival rates were statistically similar among patients with MSI-H and MSS colonic cancers. Since the former study included only patients with colon cancer, the present study aimed to assess the impact of MSI status on the outcome of early-onset compared to late-onset rectal cancer to see if this impact is similar or different from that in colon cancer.

## Patients and methods

### Study design and setting

This study was a retrospective cohort analysis of patients with rectal cancer in the US National Cancer Database (NCDB) between 2004 and 2019. The NCDB includes hospital registry data from > 1500 Commission on Cancer (CoC) accredited hospitals in the United States. The American College of Surgeons and the Commission on Cancer have not verified and are not responsible for the analytic or statistical methodology employed, or the conclusions drawn from these data by the investigator.

### Ethical considerations and reporting

Since this study was a retrospective analysis of a public database that entails de-identified patient data, institutional review board/ethics committee approval and written informed consent to participate in the study were not required. The study has been reported consistent with the Strengthening the Reporting of Observational Studies in Epidemiology (STROBE) guideline [10].

### Selection criteria

The NCDB Participant User File (PUF) was reviewed by two investigators (S.E. & N.H.), using the relevant PUF dictionary to interpret the different data variables. Patients included to the study had rectal adenocarcinoma (ICDO-3 code 8140/3, 8480–8481/3, 8490/3) with known MSI status. We excluded patients with unknown MSI status from the study.

### Data collection

The following data were collected and used for the analysis: age, sex, race, Charlson score, residence area, insurance status, clinical and pathologic TNM stage, tumor histology, grade, and size, lymphovascular invasion, perineural invasion, MSI status, systemic therapy, type of surgery, 30-day and 90-day mortality, 30-day readmission, and overall survival (OS).

### Study strategy and outcomes

The study classified patients according to the MSI status into two groups: MSI-H (patients with high microsatellite instability tumors) and MSS (patients with microsatellite stable tumors or low microsatellite instability tumors) [11]. Each group was then subdivided according to the age of diagnosis into two groups: early-onset (< 50 years) and late-onset (50 years and older). Therefore, four groups were compared regarding baseline patient and tumor characteristics, treatments, short-term outcomes, and survival. The four groups included MSI-H early-onset, MSI-H late-onset, MSS early-onset, and MSS late-onset. The primary outcome of the study was the 5-year OS whereas secondary outcomes included hospital stay, surgical margins, 30-day and 90-day mortality, and 30-day readmission.

### Addressing bias

Data used in the study were derived from a large national database to minimize sampling bias and have a representative sample. Patients were consecutively selected from the database to reduce selection bias.

### Statistical analyses

Statistical analyses were performed using EZR (version 1.55) [12] and R software (version 4.1.2) and SPSS™ (version 23). Continuous data were expressed as mean and standard deviation when normally distributed or otherwise as the median and interquartile range (IQR). A one-way ANOVA test was used to analyze continuous variables. Categorical data were expressed in the form of numbers and proportions and were analyzed using the Fisher exact test or Chi-Square test. A complete case analysis approach was used to deal with missing data. Kaplan-Meier and log-rank tests were used to detect differences in OS between the groups. *P* values < 0.05 were considered statistically significant.

## Results

**Fig. 1:**
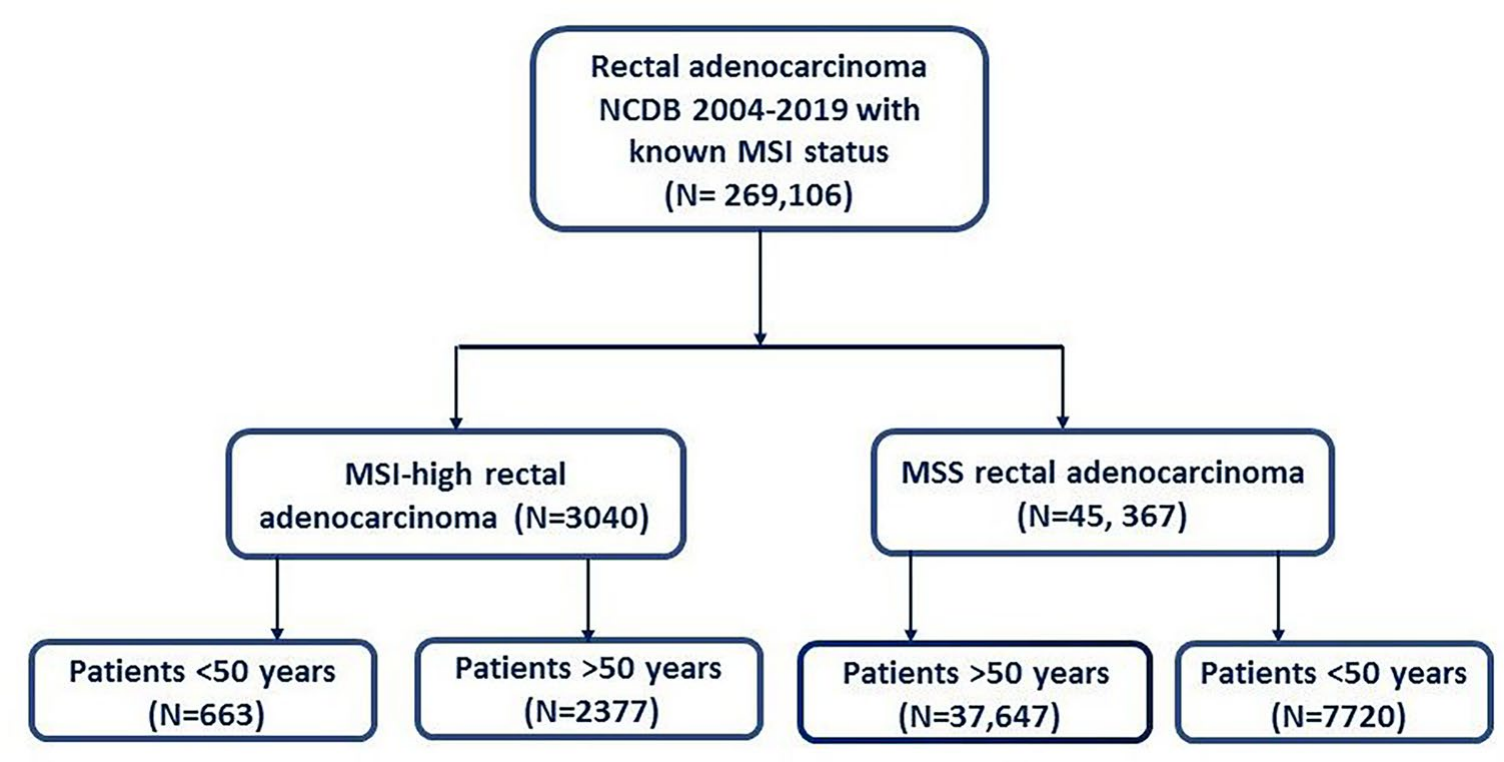
Flow chart illustrating patient selection for the study

The present study included 48,407 patients (59.9% male) with rectal cancer with a known MSI status (Fig. 1). Overall, 8383 (17.3%) patients were younger than 50 years. Some 3040 (6.3%) patients had MSI-H tumors whereas 45,367 (93.7%) had MSS tumors. Patients were classified according to MSI status and age into 4 groups: MSI-H early-onset (*n* = 663), MSI-H late-onset (*n* = 2377), MSS early-onset (*n* = 7720), and MSS late-onset (*n* = 37,647).

### Demographics

In the early-onset group, patients with MSI-H tumors had a lower mean age (41.5 vs 43 years, *p* < 0.001) than patients with MSS tumors while in the late-onset group patients with MSI-H and MSS tumors had similar mean age. The sex and race distribution of patients and insurance types were similar in the two age groups. In the late-onset group, patients with MSI-H tumors more often had a Charlson score of 0 (75.4% vs 73.3%, *p* = 0.047). Overall, more patients in the late-onset group had a Charlson score ≥ 1 than did younger patients in both the MSI-H (24.6% vs 11.3%) and the MSS group (26.4% vs 11.8%) (Table 1).

**Table 1:** Baseline characteristics and disease staging in the four groups

**Factor**	**Group**	**Early-onset**			**Late-onset**		
		**MSS (*n* = 7720)**	**MSI-H (*n* = 663)**	***P*-value**	**MSS (*n* = 37,647)**	**MSI-H (*n* = 2377)**	***P*-value**
**Mean age in years (SD)**		43.05 (5.60)	41.53 (6.90)	**< 0.001**	66.38 (10.67)	66.07 (10.68)	0.176
**Sex (%)**	**Male**	4403 (57.0)	395 (59.6)	0.205	22,770 (60.5)	1425 (59.9)	0.604
	**Female**	3317 (43.0)	268 (40.4)		14,877 (39.5)	952 (40.1)	
**Race (%)**	**White**	6371 (83.4)	559 (85.3)	0.735	32,249 (86.4)	2029 (86.1)	0.202
	**Black**	689 (9.0)	52 (7.9)		3092 (8.3)	206 (8.7)	
	**Asian**	371 (4.9)	30 (4.6)		1400 (3.7)	76 (3.2)	
	**American Indian**	57 (0.7)	4 (0.6)		153 (0.4)	7 (0.3)	
	**Other**	153 (2.0)	10 (1.5)		449 (1.2)	38 (1.7)	
**Charlson Deyo Score (%)**	**0**	6806 (88.2)	588 (88.7)	0.861	27,582 (73.3)	1792 (75.4)	**0.047**
	**1**	725 (9.4)	57 (8.6)		6999 (18.6)	421 (17.7)	
	**2**	125 (1.6)	12 (1.8)		1967 (5.2)	113 (4.8)	
	**3**	64 (0.8)	6 (0.9)		1099 (2.9)	51 (2.1)	
**Geographic residence (%)**^**a**^	**Metro**	6341 (85.1)	542 (83.9)	0.653	30,007 (82.1)	1873 (81.0)	0.089
	**Urban**	1013 (13.6)	96 (14.9)		5797 (15.9)	376 (16.3)	
	**Rural**	95 (1.3)	8 (1.2)		746 (2.0)	62 (2.7)	
**Insurance status (%)**	**Medicaid**	973 (12.8)	101 (15.5)	0.06	2451 (6.6)	166 (7.1)	0.173
	**Medicare**	284 (3.7)	26 (4.0)		18,163 (49.0)	1117 (47.9)	
	**Other**	102 (1.3)	15 (2.3)		498 (1.3)	28 (1.2)	
	**Private**	5792 (76.1)	467 (71.8)		14,871 (40.1)	935 (40.1)	
	**Not insured**	459 (6.0)	41 (6.3)		1102 (3.0)	88 (3.8)	
**Clinical T (%)**	**0**	13 (0.2)	0 (0.0)	0.058	72 (0.2)	2 (0.1)	0.591
	**In situ**	118 (1.6)	6 (1.0)		722 (2.0)	42 (1.9)	
	**1**	622 (8.5)	47 (7.4)		3365 (9.5)	207 (9.3)	
	**2**	825 (11.2)	62 (9.8)		4090 (11.6)	244 (10.9)	
	**3**	3635 (49.6)	324 (51.0)		15,697 (44.5)	991 (44.5)	
	**4**	679 (9.3)	80 (12.6)		3348 (9.5)	219 (9.8)	
	**X**	1442 (19.7)	116 (18.3)		7972 (22.6)	524 (23.5)	
**Clinical N (%)**	**0**	3312 (44.8)	273 (43.0)	0.185	19,585 (54.9)	1213 (53.8)	**0.047**
	**1**	2468 (33.4)	201 (31.7)		9547 (26.8)	610 (27.1)	
	**2**	1096 (14.8)	114 (18.0)		3957 (11.1)	235 (10.4)	
	**X**	518 (7.0)	47 (7.4)		2581 (7.2)	197 (8.7)	
**Clinical TNM (%)**	**0**	121 (1.9)	8 (1.5)	**0.03**	770 (2.6)	44 (2.4)	0.419
	**I**	1013 (15.9)	77 (14.0)		5844 (19.9)	344 (18.9)	
	**II**	1201 (18.9)	122 (22.2)		6919 (23.6)	463 (25.4)	
	**III**	2619 (41.2)	245 (44.6)		10,385 (35.4)	646 (35.4)	
	**IV**	1407 (22.1)	97 (17.7)		5423 (18.5)	327 (17.9)	
**Liver metastasis (%)**	**Yes**	584 (13.5)	37 (9.1)	**0.011**	1809 (9.7)	114 (8.7)	0.285
	**No**	3737 (86.5)	370 (90.9)		16,846 (90.3)	1190 (91.3)	
**Lung metastasis (%)**	**Yes**	230 (5.3)	13 (3.2)	0.077	817 (4.4)	53 (4.1)	0.695
	**No**	4077 (94.7)	393 (96.8)		17,816 (95.6)	1249 (95.9)	
**Bone metastasis (%)**	**Yes**	46 (1.1)	5 (1.2)	0.8	151 (0.8)	11 (0.8)	0.881
	**No**	4270 (98.9)	402 (98.8)		18,477 (99.2)	1291 (99.2)	
**Brain metastasis (%)**	**Yes**	5 (0.1)	0 (0.0)	1	34 (0.2)	4 (0.3)	0.357
	**No**	4311 (99.9)	407 (100.0)		18,591 (99.8)	1300 (99.7)	

Bold text in the *P* value column indicates statistical significance

*MSI *microsatellite instability, *MSS *microsatellite stable

^a^Metropolitan: counties in metro areas with < 250,000 to one million or more population; urban: urban population of 2500 to 20,000 or more, adjacent or not adjacent to a metro area; and rural: completely rural or < 2,500 urban population, adjacent or not adjacent to a metro area

There was a significant difference in disease stage between MSI-H and MSS tumors in the early-onset group as MSI-H tumors presented more frequently with stage III (44.6% vs 41.2%, *p* = 0.03) but less often with stage IV disease (17.7% vs 22.1%, *p* = 0.03). The pattern of metastatic disease in the early-onset group was significantly different as MSI-H tumors presented less often with liver metastasis (9.1% vs 13.5%, *p* = 0.011) compared to MSS tumors. Conversely, in the late-onset group the differences between MSI-H and MSS tumors regarding disease stage (stage III: 35.4% vs 35.4%, stage IV: 17.9% vs 18.5%, *p* = 0.419) and metastatic pattern (8.7% vs 9.7%, *p* = 0.285) were not significant (Table 1).

### Pathologic outcomes

MSI-H tumors were associated with more pathologic T3-4 stage than MSS tumors in the early-onset group (48.7% vs 45.1%, *p* = 0.004) and late-onset group (49.1% vs 41.7%, *p* < 0.001). Compared to MSS tumors, MSI-H tumors were associated with similar pN1-2 stage (34.3% vs 38.4%, *p* = 0.088) in the early-onset group, yet more pN1-2 stage in the late-onset group (31.9% vs 28.7%, *p* < 0.001). The final pathologic TNM stage showed that MSI-H tumors had stage 0-I disease less often than did MSS tumors in the late-onset group (32.6% vs 37.4%, *p* < 0.001), however, the pathologic TNM stage was similar in the early-onset group (*p* = 0.083).

MSI-H tumors were more likely mucinous carcinomas compared to MSS tumors, in both the early-onset and late-onset groups (7.5% vs 4%, *p* < 0.001). The proportion of poorly differentiated carcinomas was higher in the MSI-H group than in the MSS group in the early-onset group (18% vs 11%, *p* < 0.001) and the late-onset group (13.6% vs 10.1%, *p* < 0.001). The median number of lymph nodes harvested in the MSI-H group was higher in the early-onset group (15 vs 14, *p* = 0.081) but the opposite was found in the late-onset group (12 vs 13, *p* < 0.001). The median size of MSI-H tumors was significantly larger than MSS tumors in both groups (Table 2).

**Table 2:** Pathologic outcomes in the four groups

**Factor**	**Group**	**Early-onset**			**Late-onset**		
		**MSS (*n* = 7720)**	**MSI-H (*n* = 663)**	***P* value**	**MSS (*n* = 37,647)**	**MSI-H (*n* = 2377)**	***P* value**
**Pathologic T (%)**	**0**	337 (5.9)	34 (6.2)	**0.004**	1049 (4.3)	52 (3.0)	**< 0.001**
	**In situ**	129 (2.3)	6 (1.2)		671 (2.7)	39 (2.2)	
	**1**	813 (14.2)	68 (12.5)		4579 (18.7)	252 (14.5)	
	**2**	1385 (24.2)	127 (23.3)		5882 (24.0)	402 (23.1)	
	**3**	2267 (39.5)	212 (39.0)		8920 (36.4)	726 (41.8)	
	**4**	320 (5.6)	53 (9.7)		1307 (5.3)	127 (7.3)	
	**X**	482 (8.4)	44 (8.1)		2117 (8.6)	139 (8.0)	
**Pathologic N (%)**	**0**	2817 (49.3)	294 (53.9)	0.088	13,297 (54.8)	929 (53.9)	**< 0.001**
	**1**	1420 (24.9)	111 (20.4)		4877 (20.1)	358 (20.8)	
	**2**	769 (13.5)	76 (13.9)		2087 (8.6)	192 (11.1)	
	**X**	703 (12.3)	64 (11.7)		3990 (16.5)	243 (14.1)	
**Pathologic TNM (%)**	**0**	176 (3.5)	10 (2.1)	0.083	781 (3.7)	45 (3.0)	**< 0.001**
	**I**	1383 (27.6)	136 (28.8)		7118 (33.7)	451 (29.6)	
	**II**	909 (18.1)	105 (22.2)		4639 (22.0)	388 (25.5)	
	**III**	1741 (34.7)	154 (32.6)		5888 (27.9)	460 (30.2)	
	**IV**	810 (16.1)	67 (14.2)		2678 (12.7)	179 (11.8)	
**Tumor histology (%)**	**Adenocarcinoma**	7331 (95.0)	604 (91.1)	**< 0.001**	35,877 (95.3)	2169 (91.2)	**< 0.001**
	**Mucinous carcinoma**	305 (4.0)	50 (7.5)		1519 (4.0)	178 (7.5)	
	**Signet ring cell carcinoma**	84 (1.1)	9 (1.4)		251 (0.7)	30 (1.3)	
**Tumor grade (%)**	**Well-differentiated**	489 (8.9)	42 (8.1)	**< 0.001**	2561 (11.1)	145 (8.8)	**< 0.001**
	**Moderately differentiated**	4296 (78.4)	374 (72.3)		17,990 (77.8)	1253 (75.9)	
	**Poorly differentiated**	604 (11.0)	93 (18.0)		2330 (10.1)	225 (13.6)	
	**Undifferentiated**	90 (1.6)	8 (1.5)		257 (1.1)	28 (1.7)	
**Median number of examined lymph nodes [IQR]**		14 [3, 21]	15 [5.25, 22]	0.081	13 [0, 18]	12 [0, 18]	**< 0.001**
**Median number of positive lymph nodes [IQR]**		0[0, 2]	0 [0, 2]	0.057	0 [0, 1]	0 [0, 1]	**0.015**
**Median tumor size in mm [IQR]**		40 [25, 55]	42 [27, 60]	**0.002**	36 [22, 51]	40 [24, 52]	**0.036**
**Lymphovascular invasion (%)**	**Yes**	1313 (26.4)	106 (22.7)	0.087	4845 (21.3)	364 (23.3)	0.075
	**No**	3654 (73.6)	360 (77.3)		17,869 (78.7)	1201 (76.7)	
**Perineural invasion (%)**	**Yes**	28 (17.8)	2 (16.7)	1	175 (14.7)	10 (22.7)	0.193
	**No**	129 (82.2)	10 (83.3)		1012 (85.3)	34 (77.3)	

﻿Bold text in the *P* value column indicates statistical significance

*MSI *microsatellite instability, *MSS *microsatellite stable, *IQR *interquartile range

### Treatments

Late-onset patients with stage II-III MSI-H rectal cancers received neoadjuvant radiation therapy (58.9% vs 55.1%, *p* = 0.009) and neoadjuvant systemic therapy (40% vs 36.2%, *p* = 0.005) significantly more often than did patients with MSS tumor whereas these differences were not statistically significant in the early-onset group (*p* = 0.732 and 0.309). MSI-H tumors were treated with a longer duration of radiation therapy in the late-onset group (31 vs 24 days) but not in the early-onset group (38 vs 38 days). There were no significant differences in immunotherapy given to MSI-H and MSS tumors in both age groups.

The time from diagnosis to surgery was longer in the early-onset group than in the late-onset group. In the both age groups, MSI-H tumors were more often treated with abdominoperineal resection (APR) (18.4% vs 15.6%) and pelvic exenteration (4.4% vs 2.7%) and less often with local excision (5.3% vs 8.6%). Overall, late-onset patients underwent local excision more often than early-onset patients whereas low anterior resection was more often performed in the early-onset group. The surgical approach showed significant differences as patients with MSI-H tumors were more frequently operated on using an open approach in the early-onset (47% vs 40.3%, *p* = 0.008) and late-onset groups (44.8% vs 41.1% *p* = 0.014) (Table 3).

**Table 3:** Treatments used in the four groups

**Factor**	**Group**	**Early-onset**			**Late-onset**		
		**MSS (*n* = 7720)**	**MSI-H (*n* = 663)**	***P* value**	**MSS (*n* = 37,647)**	**MSI-H (*n* = 2377)**	***P* value**
**Radiation treatment for stage II-III disease (%)**	**No radiation given**	767 (20.5)	69 (19.4)	0.732	6303 (37.3)	351 (32.2)	**0.009**
	**Neoadjuvant**	2721 (72.7)	267 (75.0)		9296 (55.1)	642 (58.9)	
	**Adjuvant**	232 (6.2)	17 (4.8)		1211 (7.2)	89 (8.2)	
	**Neoadjuvant and adjuvant**	19 (0.5)	2 (0.6)		61 (0.4)	6 (0.6)	
	**Intraoperative**	5 (0.1)	1 (0.3)		15 (0.1)	2 (0.2)	
**Median duration of radiation days [IQR]**		38 [0, 41]	38 [0, 40]	0.690	24 [0, 40]	31 [0, 40]	0.097
**Systemic treatment for stage II-III disease (%)**	**No systemic therapy**	568 (15.3)	56 (15.5)	0.309	5366 (32.8)	293 (27.6)	**0.005**
	**Neoadjuvant**	1394 (37.6)	141 (39.1)		5915 (36.2)	424 (40.0)	
	**Adjuvant**	326 (8.8)	33 (9.1)		1531 (9.4)	115 (10.8)	
	**Neoadjuvant and adjuvant**	1423 (38.3)	131 (36.3)		3534 (21.6)	228 (21.5)	
	**Intraoperative**	0 (0.0)	0 (0.0)		11 (0.01)	0 (0.0)	
**Type of chemotherapy (%)**	**No chemotherapy**	4281 (56.5)	337 (51.9)	0.153	24,237(67.4)	1483 (65.8)	**0.032**
	**Single agent**	1068 (14.1)	102 (15.7)		5268 (14.6)	378 (16.8)	
	**Multiagent**	2160 (28.5)	203 (31.3)		6093 (16.9)	376 (16.7)	
	**Type not documented**	72 (0.9)	7 (1.1)		366 (1.0)	17 (0.8)	
**Immunotherapy (%)**	**Yes**	447 (5.8)	28 (4.2)	0.097	1314 (3.5)	67 (2.8)	0.092
	**No**	7245 (94.2)	633 (95.8)		36,197 (96.5)	2300 (97.2)	
**Median days from diagnosis to surgery [IQR]**		121[42,149]	124 [47,155]	0.091	97 [23, 144]	94 [23, 143]	0.998
**Type of surgery (%)**	**APR**	865 (15.6)	87 (18.4)	**< 0.001**	4437 (16.4)	311 (17.8)	**< 0.001**
	**LAR**	3333 (60.3)	274 (57.9)		14,983 (55.4)	929 (53.3)	
	**Local excision**	477 (8.6)	25 (5.3)		4449 (16.4)	262 (15.0)	
	**Pelvic exenteration**	152 (2.7)	21 (4.4)		604 (2.2)	54 (3.1)	
	**Pull through coloanal**	444 (8.0)	35 (7.4)		1610 (6.0)	106 (6.1)	
	**Total proctocolectomy**	163 (2.9)	28 (5.9)		551 (2.0)	60 (3.4)	
	**Proctectomy NOS**	45 (0.8)	1 (0.2)		139 (0.5)	10 (0.6)	
	**Surgery not specified**	49 (0.9)	2 (0.4)		278 (1.0)	12 (0.7)	
**Approach (%)**	**Open**	2026 (40.3)	209 (47.0)	**0.008**	9517 (41.2)	701 (44.8)	**0.014**
	**Laparoscopic**	1934 (38.5)	164 (36.9)		9218 (39.9)	598 (38.3)	
	**Robotic**	1066 (21.2)	72 (16.2)		4340 (18.8)	264 (16.9)	

﻿Bold text in the *P* value column indicates statistical significance

*MSI *microsatellite instability, *MSS *microsatellite stable, *IQR *interquartile range, *LAR *low anterior resection, *APR *abdominoperineal resection

### Short-term outcomes

The rates of conversion from minimally invasive to open surgery were similar between MSI-H and MSS patients in the early-onset (13.1% vs 9.5%, *p* = 0.087) and late-onset group (10.3% vs 9.9%, *p* = 0.681). The median hospital stay was 5 days in all groups. 30-day and 90-day mortality rates were comparable among MSI-H and MSS tumors, but early-onset patients had lower rates of 30-day and 90-day mortality than did late-onset patients (Table 4).

**Table 4:** Outcomes of the four groups

**Factor**	**Group**	**Early-onset**			**Late-onset**		
		**MSS (*n* = 7720)**	**MSI-H (*n* = 663)**	***P*-value**	**MSS (*n* = 37,647)**	**MSI-H (*n* = 2377)**	***P*-value**
**Conversion (%)**	**Yes**	286 (9.5)	31 (13.1)	0.087	1342 (9.9)	89 (10.3)	0.681
	**No**	2714 (90.5)	205 (86.9)		12,216 (90.1)	773 (89.7)	
**Surgical margins (%)**	**Positive**	488 (7.7)	52 (9.2)	0.216	2399 (7.8)	176 (9.1)	0.050
	**Negative**	5866 (92.3)	505 (90.8)		27,421 (92.2)	1759 (90.9)	
**Median hospital stay in days [IQR]**		5 [3, 7]	5 [4, 8]	**< 0.001**	5 [3, 8]	5 [3, 8]	**0.032**
**30-day mortality (%)**	**Yes**	11 (0.2)	1 (0.2)	1	451 (1.5)	22 (1.1)	0.178
	**No**	6366 (99.8)	556 (99.8)		29,246 (98.5)	1939 (98.9)	
**90-day mortality (%)**	**Yes**	36 (0.6)	4 (0.7)	0.560	892 (3.0)	48 (2.5)	0.170
	**No**	6313 (99.4)	552 (99.3)		28,694 (97.0)	1909 (97.5)	
**30-day readmission (%)**	**No readmission**	7093 (93.2)	609 (92.8)	0.307	34,814 (93.9)	2178 (93.4)	0.647
	**Planned**	120 (1.6)	6 (0.9)		457 (1.2)	30 (1.3)	
	**Unplanned**	389 (5.1)	41 (6.2)		1781 (4.8)	124 (5.3)	
	**Planned and unplanned**	10 (0.1)	0 (0.0)		35 (0.1)	1 (0.0)	
**Overall survival (%)**	**Alive**	5440 (71.9)	472 (71.8)	1	21,221 (58.1)	1376 (59.1)	0.351
	**Dead**	2131 (28.1)	185 (28.2)		15,278 (41.9)	951 (40.9)	
**Median follow-up in months [IQR]**		52.6 [33.9, 80.1]	52.3 [32.4, 78.4]	0.540	46 [24.2 72.3]	47.7 [25.6, 73]	0.061

Bold text in the *P* value column indicates statistical significance

*MSI *microsatellite instability, *IQR *interquartile range

### Overall survival

The 5-year OS rate for early-onset rectal cancer patients was significantly (*p* < 0.001) higher than that for late-onset patients, in both MSI-H (71.8% vs 59.1%, respectively) and MSS groups (71.9% vs 58.1%, respectively). The higher survival in favor of early-onset patients was demonstrated in stage I: (91.4% vs 79.4%, *p* < 0.001), stage II (80.8% vs 68%, *p* < 0.001), and stage III (71.3% vs 61.2%, *p* < 0.001), but not for stage IV disease (35.9% vs 35.5%, *p* = 1). Overall, the median OS duration of the early-onset group (139.6 months; 95%CI: 136.9–142.2) was significantly (*p* < 0.001) longer than that of the late-onset group (106.1 months; 95%CI: 104.9–107.3).

**Fig. 2:**
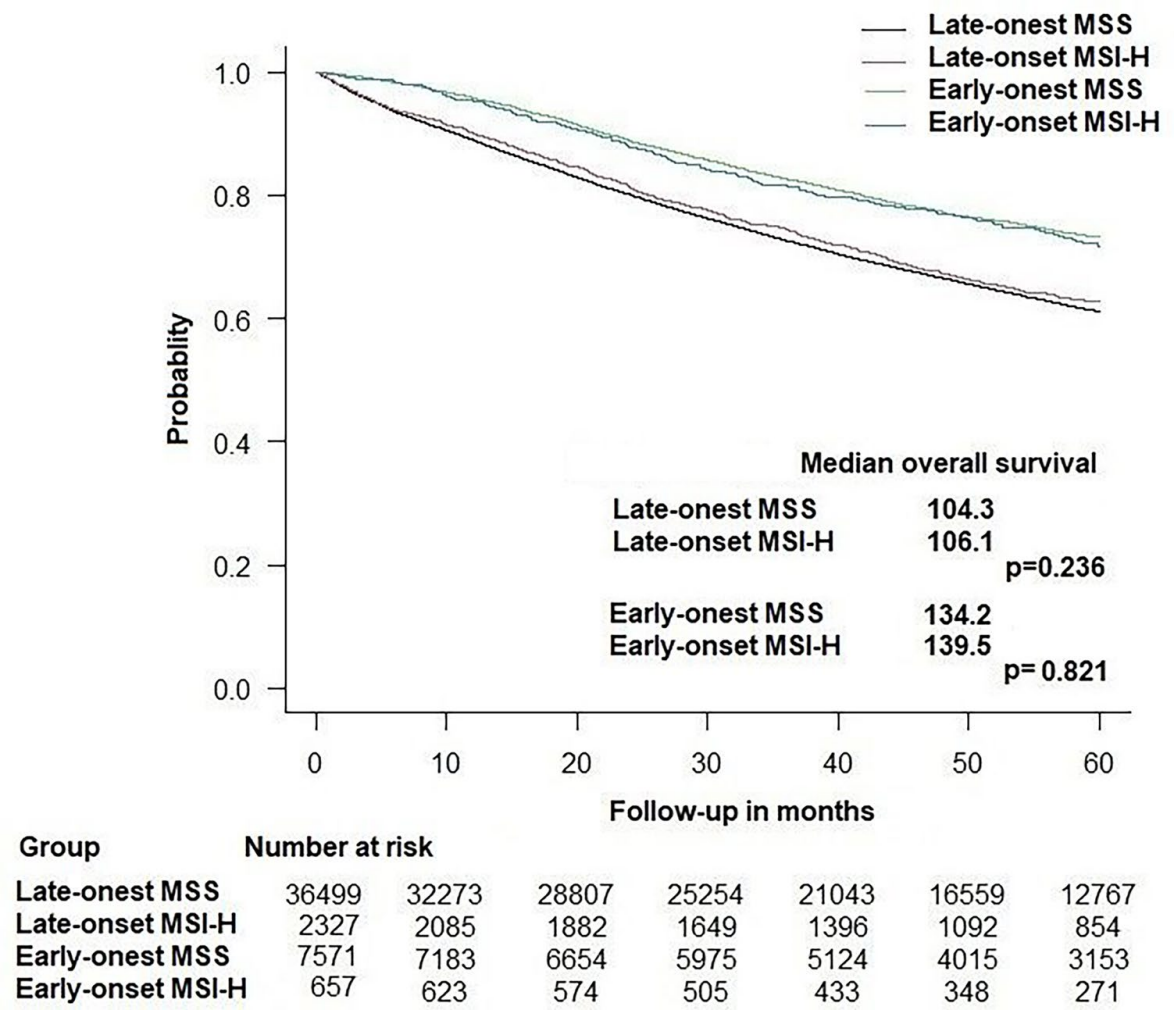
Kaplan-Meier curve showing overall survival in early-onset and late-onset rectal cancer according to MSI status. MSI, microsatellite instability

The median OS for MSI-H tumors was 108.09 months (95%CI: 101.1–116.1) and for MSS tumors was 102.31 months (95%CI: 99.8–104.8) (*p* = 0.1). When stratified by age group, the difference in OS duration between MSI-H and MSS tumors was not statistically significant for both the early-onset group (139.5 vs 134.2 months, *p* = 0.821) and the late-onset group (106.1 vs 104.3 months, *p* = 0.236) (Fig. 2). When stratified by stage, MSI-H had statistically similar OS to MSS tumors for stage I disease (117.1 vs 141.1, *p* = 0.767), stage II (118.6 vs 107.5 months, *p* = 0.179), stage III (120.5 vs 115.9 months, *p* = 0.490), and stage IV (43.6 vs 43.6 months, *p* = 0.827).

## Discussion

Microsatellite stability status has been found to significantly impact oncologic outcomes and survival of CRC [8]. Our study found that 6.3% of all rectal cancer patients were MSI-H. While MSI-H tumors account for 5–15% of all colorectal cancers [13], it has been previously shown that MSI-H rectal cancers are more infrequent than MSI-H colon cancer and may account for as low as 2% of all rectal cancers [14]. The present study found that early-onset patients had more advanced disease, received more systemic and radiation treatment, and had longer OS than late-onset tumors. The impact of MSI status on the disease stage and outcomes was not significant, except that MSI-H tumors had a lower rate of stage IV disease, particularly liver metastasis, compared to MSS tumors in the early-onset group.

The incidence of CRC in patients younger than 50 years has been rising in a concerning manner [15]. The explanation for this rising incidence is not clear and may be attributable to more exposure of young patients to risk factors of CRC including Western-style diet, obesity, and physical inactivity, all may induce genetic mutations in the epithelial lining of the colon and may alter the gut microbiota and local immunity, as highlighted in a previous study [16]. Analysis of the demographic characteristics of patients in our study showed that early-onset rectal cancer accounted for 17.3% of all patients, in line with the previous literature.

The incidence of MSI-H rectal cancer in the early-onset group was higher than that in the late-onset group (8% vs 6%), consistent with the findings of a prior study [9]. The impact of MSI status on the characteristics and demographics of patients was not remarkable. Early-onset patients with MSI-H rectal cancer were younger than patients with MSS tumors by two years on average, which may not be clinically relevant though it was statistically significant. As expected, patients with late-onset tumors had higher Charlson scores than early-onset patients, regardless of the MSI status.

Final pathologic assessments revealed that more MSI-H tumors were of T3/4 stage than were MSS tumors, in both age groups. This finding was in agreement with another study that reported a higher incidence of pT3/4 disease in CRC patients with MSI-H than in patients with MSS tumors (89% vs 74%) [17]. Early-onset patients had more nodal metastases than late-onset patients, and the incidence of nodal disease was slightly lower with MSI-H than with MSS tumors (34.3% vs 38.4%). Again, this observation was consistent with another study [17] that reported less nodal involvement with MSI-H CRC. Therefore, both studies agreed that MSI-H early-onset rectal cancers have less chance to spread to the regional lymph nodes though they tend to infiltrate deeper through the rectal wall. Early-onset patients had more stage III-IV disease than did late-onset patients which is expected given the more aggressive nature and more rapid spread of CRC in young patients [18]. The worst scenario was early-onset patients with MSS rectal cancer as more than 20% of whom presented with a metastatic disease on diagnosis, higher than early-onset patients with MSI-H cancer. The pattern of metastasis was also notable as early-onset MSI-H rectal cancer had less affinity to spread to the liver than MSS tumors.

Another interesting finding was the greater number of harvested lymph nodes in early-onset patients and patients with MSI-H tumors. It has been formerly reported that MSS CRC is more likely to be associated with sub-optimal lymph node harvest (< 12) than MSI-H tumors [17]. Other previous studies documented the same finding as Belt et al. [19] also found high lymph node yield to be related to MSI-H phenotype in stage III colon cancer. While the explanation of this observation is unclear, some authors [20] proposed that greater lymph node yield in MSI-H CRC specimen is attributable to a Crohn’s disease-like reaction.

There was a trend to give patients with early-onset rectal cancer, whether they had MSI-H or MSS tumors, more neoadjuvant radiation, and systemic therapy than late-onset patients. A recent study [21] also showed that 67% of early-onset rectal cancer patients received neoadjuvant chemoradiation therapy versus 53% of patients aged ≥ 50 years. Furthermore, we found that early-onset patients waited before surgery for 3 weeks on average longer than regular onset patients, perhaps because they received more neoadjuvant treatments. Early-onset patients were more likely to have radical operations as anterior resection than local excision, compared to late-onset patients. This observation may reflect a direction to treat younger patients with more radical resection to eradicate the more advanced disease with which they usually present.

Early-onset patients had lower short-term mortality and increased OS than did late-onset patients. The mortality rates at 30 and 90 days after surgery in the early-onset group were approximately one-fifth of those in patients above 50 years. This finding is plausible since younger patients usually have better functional status with fewer medical comorbidities that can contribute to increased short-term mortality. Although the longer OS of early-onset patients is expected, the MSI status did not have a significant association with OS in either age group. MSI-H patients had similar mean OS to MSS patients in the early-onset and the late-onset groups. This finding is in discordance with previous studies that showed a survival benefit of MSI-H CRC; however, it is consistent with a recent meta-analysis [22] that demonstrated similar hazard ratios of OS for the MSI-H and MSS groups in stage III and IV CRC. The meta-analysis concluded that MSI-H had a beneficial prognostic effect on disease-free survival, but not on OS in advanced CRC. The conclusions of this meta-analysis support our findings as 55% of the patients included in our study had stage III/IV disease. Moreover, although there is evidence on the survival benefit of immunotherapy in MSI-H CRC [23], these benefits were not demonstrated in our study owing to the low proportion of MSI-H patients who received immunotherapy in both age groups.

Compared to the findings of the REACCT collaborative study [9] on MSI status in early-onset colon cancer, our study found that patients with MSI-H early-onset rectal cancer had a similar mean age (41.5 vs 40 years) and a similar proportion of stage III disease (44.6% vs 45.9%) to patients with MSI-H early-onset colon cancer but included more male patients (59.6% vs 54.1%) and a lower proportion of signet-ring cell carcinomas (1.4% vs 10.9%), poorly differentiated/undifferentiated carcinomas (19.5% vs 28.1%), and lymphovascular invasion (22.7% vs 38%).

The present study is one of the largest analyses of the association between MSI and the outcome of rectal cancer in different age groups. The findings of this study may have clinical and research implications. MSI-H rectal cancers tend to present less often with distant metastases in early-onset but not in late-onset rectal cancer. This finding may warrant future research to investigate how and why age would affect the prognostic effect of MSI-H status. Moreover, MSI-H status was associated with an increased likelihood of nodal involvement in late-onset rectal cancers, but not in early-onset rectal cancers. The explanation of this finding is unclear and further research into the association between MSI status and age in terms of nodal involvement in rectal cancer is needed. Finally, the association between MSI status and survival was not notable in this study, whether in the early-onset or late-onset groups as the difference in OS between MSI-H and MSS tumors was less than 6 months. This finding may be explained by the higher frequency of high-grade and mucinous carcinomas and locally advanced T3/4, N1-2 tumors in the MSI-H group. Nonetheless, prospective studies are needed to assess the survival differences in rectal cancer according to the MSI status.

However, some limitations to the study should be acknowledged, including the retrospective nature of the data used in the study and the missing MSI status of many patients. Also, since data on disease recurrence were not included in the database, an analysis of the differences in disease-free survival between the age and MSI groups was not possible.

## Conclusions

In both age groups, MSI-H rectal cancers were more often mucinous and poorly differentiated carcinomas and had pT3-4 stage more often than MSS cancers. MSI-H rectal cancers tend to present less often with distant metastases and nodal involvement than MSS cancers only in early-onset, but not in late-onset rectal cancers. The association between MSI status and survival was not notable in this study, whether in the early-onset or late-onset groups.

## Data Availability

Upon reasonable request to first author.
